# Emergency Department Workload and Crowding During a Major Electronic Health Record Breakdown

**DOI:** 10.3389/fpubh.2019.00267

**Published:** 2019-09-12

**Authors:** Jens Wretborn, Ulf Ekelund, Daniel B. Wilhelms

**Affiliations:** ^1^Department of Emergency Medicine, Local Health Care Services in Central Östergötland, Linköping, Sweden; ^2^Department of Clinical Sciences Lund, Emergency Medicine, Faculty of Medicine, Lund University, Lund, Sweden; ^3^Department of Medical and Health Sciences, Faculty of Health Sciences, Linköping University, Linköping, Sweden

**Keywords:** electronic health records, breakdown, crowding, workload, emergency department

## Abstract

**Background:** Emergency Departments (EDs) today rely heavily on Electronic Health Records (EHRs) and associated support systems. EHR updates are known to be associated with adverse events, but reports on the consequences of breakdowns in EDs are lacking.

**Objectives:** To describe the effects on workload, occupancy, patient Length Of Stay (LOS), and admissions at three EDs (a regional trauma center, a community hospital and a rural community hospital) during a 96 h period of EHR downtime, of which 48 h represented an unexpected breakdown.

**Methods:** Assessments of workload, on a scale from 1 (no workload) to 6 (very high workload), were obtained from all staff before, during and after the downtime period. Occupancy, LOS and hospital admissions were extracted from data recorded in the fallback system at each ED during the downtime, and compared with the period before and after (uptime).

**Results:** Workload increased considerably at two EDs during the downtime whereas the third ED lacked resources to assess workload due to the breakdown. The proportion of assessments **≥**4 were 28.5% during uptime compared to 38.4% during downtime at the regional trauma center ED (difference 9.9%, *p* = 0.006, 95% CI 2.7–17%), and 22.9% compared to 41% at the rural community ED (difference 18.1%, *p* = 0.0002, 95%CI 7.9–28.3%). Median LOS increased by 19 min (3:56 vs. 4:15, *p* < 0.004) at the regional trauma center ED, by 76 min (3:34 vs. 4:50, *p* < 0.001) at the community ED and was unaltered at the rural community ED (2:47 vs. 2:51, *p* = 0.3) during downtime. Occupancy increased significantly at the community ED (1.59 vs. 0.71, *p* < 0.0001). Admissions rates remained unchanged during the breakdown. Fallback systems and initiatives to manage the effects of the breakdown differed between the EDs.

**Conclusions:** EHR downtime or unexpected breakdowns increased staff workload, and had variable effects on ED crowding as measured by LOS and occupancy. Additional staff and digital fallback systems may reduce the effects on ED crowding, but this descriptive study cannot determine causality.

## Introduction

Many healthcare systems today rely heavily on Electronic Health Records (EHRs). Four domains have been suggested as key EHR constituents: Clinical data repository, clinical decision support systems, Computerized Physician Order Entry (CPOE), and Electronic Medication Assistance Record (EMAR) (1). In Sweden and large parts of Europe and the United States, emergency medicine providers use these integrated EHRs (2, 3).

Legislation dictates strict terms for EHR access to ensure patient privacy (4), but is generally less detailed concerning the responsibility for EHR reliability and to have routines for situations when the EHR malfunctions (5). Updates in clinical information systems are known to be associated with adverse events (6), but there is a surprising paucity of descriptions of major EHR breakdowns in the medical literature.

In modern hospitals, the Emergency Department (ED) is the nexus for patient inflow. The complex combination of high inflow and patients with high acuity illnesses frequently causes ED crowding. ED crowding has become a common problem internationally and is associated with increased mortality and decreased quality of care (7–9).

So what happens in modern ED when the EHR breaks down, not in a short annoying glitch or for a few tiresome hours, but for several days and without a clear timetable for resolution?

We recently experienced such a breakdown in a whole Swedish county with 457,000 inhabitants and three emergency hospitals, one of which is an academic teaching hospital and regional trauma center with a total catchment area of three counties and ~1,050,000 inhabitants. The breakdown was unexpected, coming in the wake of a planned EHR software update, which had been scheduled to last for 44 h. However, the breakdown extended the downtime to 96 h. The breakdown happened during a pre-planned research study on ED workload which included staff assessments of workload and collection of extensive administrative data. Assessments were scheduled closely before, during and after the planned update to gauge the effects of a new EHR version on the workload. Instead, however, we got data on the longest and most extensive downtime of an EHR system in an ED described so far.

Our objective with this study was to describe the effects on staff workload, ED crowding and hospital admissions at three EDs during the 96 h period of regional EHR downtime. ED crowding was measured by ED Length of Stay (LOS) and ED occupancy which have frequently been shown to be a measure of crowding (10).

## Methods

### Study Design

This was a prospective observational study on workload and crowding in three EDs during a major planned update in the EHR system. The study was approved by the regional ethics review board in Linköping (permit 2017/371-31).

### Study Setting (and Selection of Participants)

Characteristics of the three participating emergency departments, one regional trauma center ED (RTC), one community hospital ED (CH1), and one rural community hospital ED (CH2), are found in Table 1. The EHR used in all hospitals covers the four domains described by DesRoche et al. (1). All included EDs used the same documentation routine during normal practice. This routine consisted of a single monitoring/prescription paper sheet that was used for primary documentation of vital signs and urgent prescriptions of tests and medications. All other information, such as patient care events, acuity according to triage/monitoring guidelines and other process-related information were directly documented and presented in the EHR. Subsequently, all information from the ED visit was compiled in the EHR, including the information primarily documented on paper. A separate CPOE system was used for radiology referrals and laboratory testing, but with full integration into the EHR user interface.

**Table 1:** Hospital characteristics.

	**Linköping University Hospital**	**Norrköping Community Hospital**	**Motala Community Hospital**
Type	Academic Tertiary Care Center	Urban Community Hospital	Rural Community Hospital
Hospital beds	600	310	100
ED beds	38	29	15
Annual ED visits	45,000	45,000	25,000
Admission rates	22%	24%	19%
Staffing	EM consultants and residents. interns	EM residents, IM/surgical/orthopedic residents, interns	EM residents, surgical consultants, IM residents, interns

*EM, emergency medicine; IM, internal medicine*.

The manual documentation routine described above was used as the basic backup system for documentation in the event of an EHR breakdown, and was used in all EDs during this study. The RTC ED also used a digital ledger to keep track of patient processes in the ED. This digital ledger was implemented as a local web application where all providers in the ED could update processes-related information about patients in the ED. This digital ledger required functional computers, and a local network, and information was limited to process data in the ED. At the RTC, a whiteboard was also available as a secondary alternative to the digital ledger. The CH2 ED used a centrally located whiteboard for the same purpose, whereas CH1 did not have any backup or substitution for the process information registry in the EHR. The duration and anticipated effects of the scheduled downtime for the pre-planned software update was known in all emergency departments for more than a month in advance.

### Data Collection

We prospectively collected workload assessments on paper questionnaires from all health care staff at RTC and CH2 at 5 pre-specified time points each day. Data were collected between October 30 and November 19, 2017. The planned update and subsequent breakdown happened on November 10 and lasted throughout November 15. We made no distinction between planned and unplanned downtime in the analysis since backup systems and staffing was unchanged at all sites for these periods. There was a short period of ~12 h of intermittent functioning between the planned and unplanned downtime. This period was considered as uptime in the analysis. Workload assessments, patient to ED bed ratio (occupancy), patient ED (LOS), and number of hospital admissions for the total downtime period (planned and breakdown) were compared with the period when the system worked (uptime). Due to unforeseen resource limitations related to the breakdown, no workload assessments were collected in CH1.

Assessments were made on an ordinal Likert scale (11) graded from 1 to 6 with anchors at 1 (no workload) and 6 (very high workload) as previously described (12). No consensus definition of harmful workload exists in Swedish EDs, but based on our previous study, an assessment score of **≥**4 was used as the cutoff for high workload (12). ED LOS was documented on paper (CH1, CH2) or on a digital spreadsheet (RTC) and entered into the standard EHR (Cambio Cosmic R8.1, Cambio Healthcare Systems AB, Sweden) after the breakdown. Data were then collected retrospectively from the EHR to calculate occupancy, LOS, admission rates and number of visits.

### Statistical Analysis

Proportions of workload assessments and admitted patients were compared with chi-square statistic. Means were compared using student's *t*-test. Medians were compared with the Kruskal-Wallis test. Data was imported from comma-separated text files into Pandas dataframes (13) and analyzed with computer scripts in the Python programming language using the scipy library for statistical calculation (14, 15).

## Results

**Figure 1:**
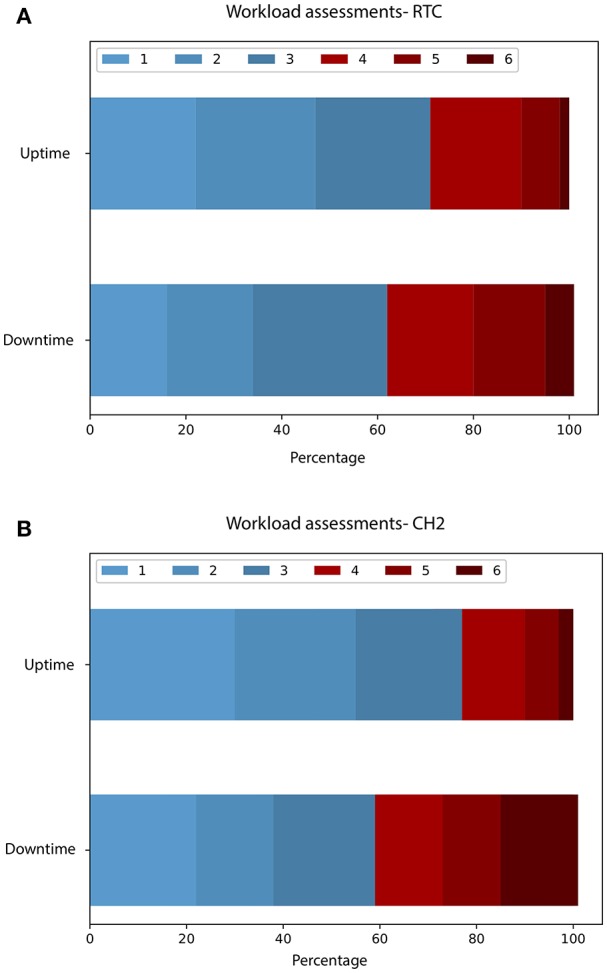
**(A,B)** Distribution of workload assessments.

A total of 1,255 and 708 assessment were collected at the RTC and CH2 ED, respectively, of which 211 (17%), and 100 (15%) during downtime. The CH1 ED lacked resources to measure workload due to the breakdown. Mean workload was lower during uptime compared to downtime (2.71, SD 1.31 vs. 3.16, SD 1.44, difference 0.45, *p* < 0.0001) at RTC ED. The same pattern was observed in CH2 ED, with mean workload being lower during uptime compared to downtime (2.50, SD 1.37 vs. 3.23, SD 1.73, difference 0.73, *p* < 0.0001). The proportion of workload assessments **≥**4, representing high workload, was clearly higher during the downtime for RTC (28.5 vs. 38.4%, difference 9.9%, *p* = 0.006, 95%CI 2.7–17%) and CH2 (22.9 vs. 41%, difference 18.1%, *p* = 0.0002, 95%CI 7.9–28.3%). Figure 1 Of note, the differences were similarly substantial regardless of assessment cutoff 2–6 for both EDs.

Median LOS increased significantly in RTC and CH1, but not in CH2. Mean occupancy was significantly higher at CH1 but not in RTC or CH2. Inflow of patients measured as day census and patients per hour was similar for all hospitals during both uptime and downtime. There was no significant difference in percentage of patients being admitted at any of the study sites (Table 2).

**Table 2:** ED process data during EHR uptime and downtime.

	**Uptime**	**Downtime**	
**Linköping University Hospital (RTC)**			
LOS (median)	03:56:30	04:15:48	***p*** **< 0.004**
Total visits	1875	458	
Occupancy	0.42	0.48	*p* = 0.53
Visits/hour, median (1-3 quartile)	4 (2–7)	4 (3–8)	*p* = 0.39
Hospital admission rate	26.4%	24.2%	*p* = 0.37
**Norrköping Community Hospital (CH1)**			
LOS (median)	03:34:02	04:50:32	***p*** **< 0.0001**
Total visits	1976	409	
Occupancy	0.71	1.59	***p*** **< 0.0001**
Visits/hour, median (1-3 quartile)	5 (2–8)	4 (2–6)	*p* = 0.15
Hospital admission rate	28.0%	25.2%	*p* = 0.27
**Motala Community Hospital (CH2)**			
LOS (median)	02:47:11	02:50:48	*p* = 0.24
Total visits	867	198	
Occupancy	0.45	0.47	*p* = 0.73
Visits/hour, median (1–3 quartile)	2 (1–4)	2 (1–4)	*p* = 0.61
Hospital admission rate	23%	24.7%	*p* = 0.66

*The bold value indicates statistically significant, p < 0.05*.

Extra staffing in response to the breakdown differed between the study sites. At the RTC, a complete extra team (EM physician, nurse, and enrolled nurse) was added. The CH1 ED added an extra EM resident daytime (08:00 to 21:00) and the CH2 ED had one extra physician allocated solely for triage during the breakdown between 08:00 and 21:00.

## Discussion

In this paper, we describe the effects on staff workload and ED crowding of an unexpected breakdown of the EHR occuring in the wake of a planned EHR update in a regional healthcare system in southern Sweden.

The unexpected breakdown of the EHR resulted in a substantial increase in perceived workload at the two hospitals where this was recorded. At one hospital, the workload was judged so high due to the breakdown that it was not deemed safe to use resources to collect workload data. Patient inflow was unchanged before, during and after the breakdown, but ED crowding, as measured by patient LOS and occupancy, was substantially increased at the two larger EDs. Interestingly, LOS was not affected at the smallest, rural hospital. Specifically, differences in turnover time between the EDs were reflected in the occupancy levels, which were doubled in CH1, slightly higher at RTC, and unchanged in CH2. We do not have data on patient acuity during the downtime, but we saw no differences in percentage of patients being admitted to any of the hospitals. This supports our interpretation that the EHR breakdown increased the level of workload and crowding in the ED. Since this breakdown occurred in conjunction with a planned update, where staff and administration were familiar with and had recently used backup routines, the size of the effect could potentially be even larger in a setting which has not recently been primed to such circumstances.

Although our study design does not allow for causality analysis, we suspect that different initiatives to manage the situation may have contributed to the different effects on LOS and occupancy. Normally, the EHR provides a digital ledger of all patients present in the ED, including acuity and plan, which may assist in managing patient flow. Throughout the downtime, this lack of overview was compensated for by a digital spreadsheet at the RTC and a centrally located whiteboard at CH2. At CH1, there was no systematic fallback system, which might explain the substantial increase in ED LOS and occupancy in that ED. Also, the small size and compact design of the CH2, which gives staff good visual overview, may have made it less dependent on the EHR patient ledger. Further, the CH1 ED had no systematic strategy for ED flow management or extra staff. This suggests that extra staff and (even rudimentary) digital replacement systems may increase the resilience of an ED to EHR breakdown. Even though the total downtime was 96 h, the period is too short to draw any further conclusions regarding the effects on patient care. However, we know from previous studies that crowding is associated with decreased quality of care in the ED (7).

The reason for the breakdown turned out to be lack of server capacity to run the new version of the EHR. This problem could most likely have been prevented, had a full-scale test of the new EHR been carried out. Such measures were suggested by clinical staff in anticipation of the update but rejected with lack of resources given as the main reason. We would, however, strongly recommend any organization planning major updates to demand full scale testing prior to the launch of a new EHR version. Preferably, there should also be a technical solution for a quick rollback in case of major technical problems. Should a general EHR breakdown still occur, despite these precautions, there should be an explicit plan for compensatory measures, which may include increased staff and a redundant system for basic digital support to record events critical to patient management. As a last resort, an alternative method independent of electricity and digital infrastructure, such as paper-based records and a whiteboard to track patients and basic logistics, should also be available and detailed in a standard operating procedure known to all staff. Since staff workload will be expected to increase substantially, we also suggest measures to counteract staff fatigue and related patient safety concerns.

On a general level, our experience provides some interesting perspectives on the vulnerability and preparedness of modern healthcare systems to a range of unexpected events. There is a clear discordance between, on the one hand, frequent reports in the media about problems concerning the safety and functionality of EHRs and, on the other hand, a relative paucity of scientific literature on the topic. Lack of unified, central reporting of EHR reliability issues in Sweden, as well as internationally, also precludes an objective analysis of the trend for this type of events. Previous studies mainly focus on health record security breaches which, reportedly, affected as many as 176.4 million US patient records in the period 2010-2017 (16). Lack of access to patient records at a time of critical illness, such as in an EHR breakdown in the ED, may however pose an even greater and more immediate risk to patient safety. To some extent, breakdown events could possibly be predicted by simulation prior to updates, such as the event covered in this report. A long-term breakdown of the EHR affecting a whole regional healthcare system will, however, most likely remain an extremely rare event. Such events do not easily lend themselves to forecasting using standard statistical models for risk assessments, since they constitute extreme outliers (17). Thus, the next event causing the EHR to fail may not be easily predicted and could very well-depend on some other, hitherto unknown, cause. In addition to increasing system robustness which, by design, will be reactive to either previous events or outlier-insensitive forecasting (17), building redundancy may be used as an additional strategy to decrease sensitivity to future adverse events. This also applies to healthcare and its delivery in a general sense. A simple example of redundancy in relation to the EHR, as detailed in the description of this event, is the use of a separate digital fallback system or a whiteboard to keep track of patients and events critical to the ED care. Because of the difficulty to predict the cause of the next breakdown, the generic key feature to build redundancy will be to decrease system-interdependencies.

In summary, the breakdown that we studied highlights that organizations should strive to increase resilience to malfunction in individual support systems by improving both robustness and building redundancy, since extreme adverse events will recur and, by nature, be very difficult or even impossible to predict and specifically counteract. By doing so, healthcare organizations will be less fragile in facing unknown future challenges.

## Limitations

All administrative data from the breakdown period was collected on paper (CH1 and CH2) or in an electronic spreadsheet-based backup system (RTC), which confers a risk of system-dependent differences in registration between uptime and downtime. Also, transcription errors could have occurred during the retrospective transfer of data from the backup systems to the EHR when it was working again.

Since this was an observational study, causal relation between increased workload, crowding and the EHR breakdown could not be established. Consequently, there may have been unmeasured confounding variables that contributed to the differences. However, given the uncontrolled nature of a large scale EHR breakdown, it would not have been practically feasible or ethically acceptable to study this phenomenon in a conventional, controlled way.

## Conclusion

This is, to the best of our knowledge, the first report on the effects of a major ED EHR breakdown. The downtime resulted in a substantial increase in workload, but the effect on crowding as measured by ED LOS and occupancy was variable. Preparedness and inbuilt redundant routines to counteract the effects of EHR downtime are most likely key elements in managing unexpected breakdowns. This scenario should be considered by all EDs using EHRs. We strongly encourage researchers and healthcare management to report large scale EHR breakdowns since more data on the extent of the problem is needed.

## Data Availability

The datasets generated for this study are available on request to the corresponding author.

## Author Contributions

DW conceived the study and drafted the manuscript. DW and JW designed the trial. DW and UE obtained the research funding. DW supervised the conduct of the trial. JW collected and managed the data, with assistance of DW and UE. All authors contributed substantially to its revision. JW takes responsibility for the paper as a whole.

### Conflict of Interest Statement

The authors declare that the research was conducted in the absence of any commercial or financial relationships that could be construed as a potential conflict of interest.
